# Microglia autophagy in ischemic stroke: A double-edged sword

**DOI:** 10.3389/fimmu.2022.1013311

**Published:** 2022-11-16

**Authors:** Li Peng, Guangqiang Hu, Qianfang Yao, Jianming Wu, Ziyang He, Betty Yuen-Kwan Law, Guishan Hu, Xiaogang Zhou, Junrong Du, Anguo Wu, Lu Yu

**Affiliations:** ^1^ Sichuan Key Medical Laboratory of New Drug Discovery and Druggability Evaluation, School of Pharmacy, Southwest Medical University, Luzhou, China; ^2^ Luzhou Key Laboratory of Activity Screening and Druggability Evaluation for Chinese Materia Medica, School of Pharmacy, Southwest Medical University, Luzhou, China; ^3^ Department of Medicine Imaging, School of Clinical Medicine, Southwest Medical University, Luzhou, China; ^4^ Department of Anatomy, School of Basic Medical Sciences, Southwest Medical University, Luzhou, China; ^5^ State Key Laboratory of Quality Research in Chinese Medicine, Macau University of Science and Technology, Macao, Macao SAR, China; ^6^ Key Laboratory of Drug Targeting and Drug Delivery Systems of Ministry of Education, Department of Pharmacology, West China School of Pharmacy, Sichuan University, Chengdu, China; ^7^ Department of Chemistry, School of Basic Medical Sciences, Southwest Medical University, Luzhou, China

**Keywords:** ischemic stroke, microglia, autophagy, neuroinflammation, therapy

## Abstract

Ischemic stroke (IS) is one of the major types of cerebrovascular diseases causing neurological morbidity and mortality worldwide. In the pathophysiological process of IS, microglia play a beneficial role in tissue repair. However, it could also cause cellular damage, consequently leading to cell death. Inflammation is characterized by the activation of microglia, and increasing evidence showed that autophagy interacts with inflammation through regulating correlative mediators and signaling pathways. In this paper, we summarized the beneficial and harmful effects of microglia in IS. In addition, we discussed the interplay between microglia autophagy and ischemic inflammation, as along with its application in the treatment of IS. We believe this could help to provide the theoretical references for further study into IS and treatments in the future.

## 1 Introduction

Stroke is the most common serious manifestation of cerebrovascular disease and ischemic stroke (IS) accounts for about 87% of all strokes (1). IS can severely affect the human well-being, thereby causing significant socio-economic problems in the world. It is characterized by a sudden interruption of blood flow to localized brain tissue, subsequent irreversible neuronal damage, and deficits in neurological functions (2). The pathophysiological mechanism of IS is quite complex, involving oxidative stress, inflammatory responses, excitotoxicity, Ca^2+^ overload and other mechanisms. Among them, microglia-mediated inflammatory response is an important pathological link that should not be overlooked (3). Microglia are highly specialized tissue-resident macrophages in the central nervous system (CNS) and are found in the parenchyma (4). Like a double-edged sword, microglia, following the incident of cerebral ischemia, exert pro-inflammatory and anti-inflammatory effects in different stages.

Autophagy plays a vital role in leveling the pros and cons of microglial inflammatory responses. It is a self-protective mechanism that exists generally in eukaryotic cells, which affects the renewal of cell metabolism and energy, maintains cellular homeostasis, and performs direct or indirect role in regulating cell survival and death. Under normal nutrient-rich conditions, autophagy is maintained at low basal levels. However, under various stressful conditions such as ischemia and hypoxia, oxidative stress, and nutrition deprivation, autophagy can degrade and recycle anomalous cytoplasmic components, including long-lived proteins, insoluble protein aggregates, and superfluous or damaged organelles to satisfy cellular needs and to promote cell survival (5). Microglia autophagy takes part in several interactive roles in post-stroke inflammatory responses. After IS, the specific regulatory mechanism of microglia autophagy and its mediated neuroinflammation form a complex molecular signal transduction network. Therefore, targeting microglia autophagy may provide new strategies in the clinical treatment of IS.

In this review, we presented how microglia exhibit both beneficial and harmful impacts in IS. We also discussed the microglia autophagy functions as both a “brake” and “accelerator” in ischemic inflammatory responses, and reviewed how therapeutic candidate target microglia autophagy in ischemic inflammatory responses, which could provide a new hope for the treatments of IS.

## 2 Ischemic stroke

**Figure 1:**
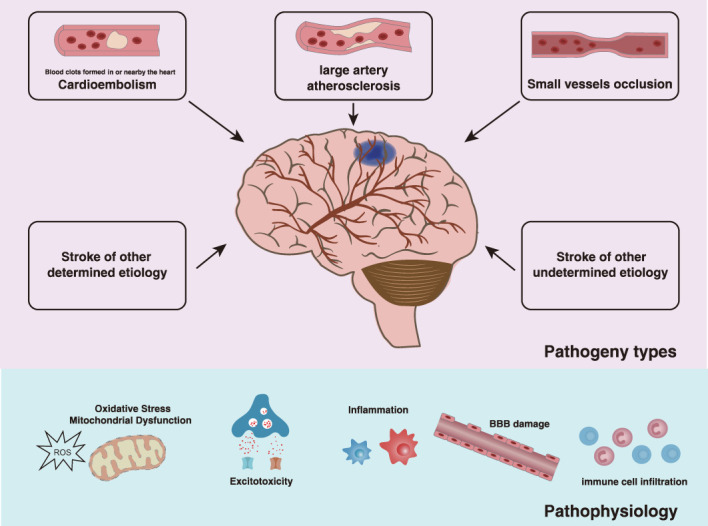
Types and effects of IS. According to the TOAST etiology classification, the etiology of IS is classified as LAA, CE, SVO, and other determined etiology or undetermined etiology. As the disease progresses, there is a reversible loss of tissue function followed by irreversible neurological deficits due to insufficient blood supply to the brain tissue, which is manifested by the ischemic core of irreversibly damaged tissue surrounded by the ischemic penumbra of hypoperfused but potentially salvageable tissue. The pathophysiological processes following IS are complex, involving energy failure, acidosis, loss of cell homeostasis, excitotoxicity, oxidative stress, activation of glial cells, inflammation, and disruption of the blood-brain barrier with infiltration of leukocytes.

IS is a rapidly increasing global burden of disease and disability of an aging population. Global burden of disease study in 2019 (GBD 2019) showed that stroke remained the second-leading cause of death and the third-leading cause of death and disability combined on a global-scale. According to the Trial of Org 10172 in Acute Stroke Treatment (TOAST) classification system, IS is divided into 5 subtypes: stroke due to either large artery atherosclerosis (LAA), cardioembolism (CE), small-vessel occlusion (SVO), and stroke of other determined etiology or of undetermined etiology (6, 7). Due to inadequate blood supply to the brain tissue, there is first a reversible loss of tissue function, which, if the disease continues to progress, may lead to subsequent irreversible neuronal damage and neurological deficits. The time from the onset of symptoms to the onset of irreversible tissue damage depends on the magnitude and duration of the decrease in cerebral blood flow; as the cerebral blood flow decreases by approximately 50%, the patient remains asymptomatic. As cerebral blood flow decreases further, reversible neuronal dysfunction causes symptoms of focal cerebral ischemia. Once the blood flow resumes quickly enough, neuronal function recovers without infarction, that is, a transient ischemic attack occurs. When the ischemia caused by a reduced blood flow lasts long enough, the brain tissue develops an irreversible infarction, leading to the pathophysiology of an IS event including energy failure, acidosis, loss of cell homeostasis, excitotoxicity, oxidative stress, activation of glial cells, inflammation, and disruption of the blood-brain barrier (BBB) with infiltration of leukocytes (8–10). This process manifests in brain tissue as an ischemic core of irreversibly damaged tissue surrounded by an ischemic penumbra of hypoperfused but potentially salvageable tissue (**Figure 1**) (11). Recent clinical advance has shown that IS affected women to a greater degree than men, and the women demonstrated worse outcomes compared to men following IS (12). Unfortunately, since the outbreak of the COVID-19 pandemic, a substantial proportion of COVID-19 patients had documented thrombotic complications and IS (13). Currently, the main therapeutic strategies available for the management of patients with IS are reperfusion - aiming for neuroprotection and neurorecovery (14). Pharmacological or mechanical reperfusion therapies are the most effective treatments with a favorable prognosis in 50-70% of cases during the acute phase of IS. These kinds of therapies include intravenous thrombolysis, endovascular therapies, and bridging intravenous-intra-arterial therapies, though endovascular therapies are commonly referred to as intra-arterial thrombolysis and mechanical thrombectomy (15–17). However, reperfusion can result in serious secondary brain tissue injury such as neuroinflammation. The modulation of microglia autophagy and inflammation has been proven to be a potential therapeutic target in the increasing trend of cerebral ischemia/reperfusion therapy studies as highlighted in this review. As for the prevention and control of IS, primary prevention of first stroke such as lifestyle modifications, management of risk factors including hypertension, diabetes mellitus and lipid disorders, antiplatelet and anticoagulation is crucial. Secondary prevention of recurrent strokes, especially carotid surgery in selected symptomatic patients, closure of patent foramen ovale (PFO) after cryptogenic stroke, and treatment of intracranial stenosis is of high priority (18). Moreover, nontraditional risk factors such as genetic predisposition have been emphasized as factors that may play an important role in the etiology of IS (19–21).

### 2.1 Microglia in ischemic stroke

#### 2.1.1 Microglia

Microglia are the only highly specialized tissue-resident macrophages localized in the parenchyma of the CNS. They are derived from primitive myeloid progenitors arising in the first wave of macrophage formation during primitive hematopoiesis before embryonic day 8 in in the yolk sac, and bone marrow–derived myeloid precursors do not contribute to the microglia pool (4, 22, 23). Microglia account for approximately 5-20% of the total glial cell population and 5-15% of all brain cells (24–26). Generally, the number of microglia in the adult brain varies depending on the regions and is maintained by the slow homeostatic proliferation of pre-existing mature microglia *in situ* in a random clonal fashion, ranging from 5% in the cerebral cortex and corpus callosum to 12% in the substantia nigra, and the hippocampus, basal ganglia, and substantia nigra are more populated by microglia compared with those in the nervous tracts, cerebellum, and brain stem (27–29). According to the microglial variations in morphology depending on their locations, three categories of microglia are broadly classified; namely compact cells (i.e., round amoeboid cells) found exclusively in sites lacking a BBB, longitudinally branched cells found in fiber tracts and radially branched cells (i.e., ramified cells) found throughout the neuropil (30, 31).

As the most abundant brain-resident mononuclear phagocytes in the immune system of the CNS, the prominent physiological function of microglia is to exert phagocytosis in regulating brain development, maintaining the stability of the CNS and participating in injury repair such as removal of damaged cellular debris, microbes, protein aggregates, and other particulate and soluble antigens that may cause harm to the CNS (22, 32–34). For instance, microglia play significant roles in synaptic pruning regulated by astrocyte and neuron-produced IL-33, and trogocytosis (a selective partial phagocytosis) restricted to presynaptic boutons and axons (27, 35–37). Moreover, this mechanism causes the phagocytosis of some myelin sheaths in a neuronal activity-regulated manner to modify developmental myelination targeted by oligodendrocytes and englobes neuronal precursor cells to control their relative abundance during development (38, 39). Microglia are also crucial in the formation of the vascular network. Before astrocyte invasion and the onset of vasculogenesis, microglia are first detected in the human retina at 10 weeks of gestation, involving in the onset of vasculogenesis in this period before the development of the retinal vasculature and in actively sprouting blood vessels (40, 41). Depletion of resident retinal microglia was found to reduce developmental vessel growth and density, but was restored by intravitreal microglial injection (42).

In many physiological and pathological processes, microglia can produce and secrete multiple factors such as chemokines, cytokines, growth factors and inflammatory factors, and simultaneously, express a wide range of immune receptors and receptors for neurotransmitters. As an example, insulin-like growth factor-1 (IGF-1), an important pro-neurogenic factor primarily produced by microglia, can influence oligodendrogenesis instigation, stimulate neurogenesis and mediate the neuroprotective responses (43–45). In addition, microglia contribute majorly in the production of growth factor transforming growth factor-beta (TGF-β), which promotes cerebrovascular remodeling following a focal ischemic insult and mediates proliferation, differentiation, and maturation of neurons and glial cells in neurodegenerative diseases (46, 47). Furthermore, the physiological activities of microglia are known to be regulated by the extracellular TGF-β. TGF-β could function as a master cytokine for microglial subpopulation by modulating microglial homeostatic properties (48, 49). By inhibiting microglial cytotoxic activation and phagocytic function, TGF-β protects the neurons against injury and neurodegeneration (47, 50, 51). CD200R, expressed only on the myeloid lineage cells including microglia, binds with CD200 to form a key immunoregulatory signal that constrains microglial activation and hinders microglia exhibiting an exaggerated or prolonged inflammatory response to subsequent immune challenges (52–54). In rat models of middle cerebral artery occlusion (MCAO), CD200-CD200R axis reduces microglial activation and levels of inflammatory cytokines, thus decreasing the infarct volume, ameliorating behavioral deficits and increasing the therapeutic recovery potential after hypoxic-ischemic brain injury (55, 56). Emerging genetic and functional evidences suggests that microglia play an important role in CNS disorders, encompassing brain inflammation, ischemia, hypoxia, stroke, and degenerative CNS diseases such as Alzheimer’s disease (AD) and Parkinson’s disease (PD).

#### 2.1.2 Microglial activation and inflammatory response

Microglia are diverse, dynamic, and heterogeneous; they have different subtypes due to intrinsic features, and each microglial subtype may respond or not to the stimulus by expanding and/or changing its morphology and gene expression to assume a specific activation state in response to an environmental cue (57, 58). Although the definition of microglia subtypes is still controversial, the widely described subtypes of microglia can be mainly divided into six categories: Satellite microglia, Keratan sulfate proteoglycan (KSPG)-microglia, Microglia supporting neurogenesis, Hox8b- microglia, Cd11c-microglia, and Dark microglia (22, 59, 60). Utilizing single-cell RNA sequencing, Li et al. identified at least six transcriptionally distinct microglial subsets MG1-6 in IS-aged mouse brains, among which MG6 is a putative stroke-specific subtype. Resting MG1 and MG2 were reduced and transferred to different microglial subtypes after stroke, primarily MG5, the predominant microglial subset in stroke, and MG6, a specific microglial state that manifests solely after stroke and has a unique “neutrophil-like” character (61). A unique microglial subtype, Cd11c+, with neurodegenerative disease-associated microglial characteristics, is detectable in the degenerative thalamus of mice after permanent occlusion of the left middle cerebral artery, implying a neuroinflammatory relationship between stroke and other neurodegenerative illnesses (62). By integrating single-cell and bulk RNA sequencing, the researchers discovered that microglia at the acute stage of IS in the mouse cortex penumbra area were prevalent and exhibited polarization and differentiation in two different progression trajectories. Among the 14 microglial subclusters, subclusters 3, 4, 9, and 10 were characterized by inflammation-related genes and pathways enriched by hypoxia, TNF-, IL-6-, and IL-2, whereas subclusters 6 and 8 were primarily enriched in the Kras signaling pathway and lacked overexpression of characteristic genes such as Arg1 and Ym1, implying that these two groups of subclusters may represent different activation states and sequences of activation states of microglia in the early stages of IS (63). Currently, there is insufficient research on microglial subtypes in IS. The function of microglial subtypes in IS merits further investigation, with a focus on variations in subtypes on the time course, which may necessitate long-term monitoring to provide new insights into the involvement of microglia in IS pathogenesis.

The neuroinflammatory state of the CNS plays a crucial part in physiological and pathological conditions after cerebral ischemia or other CNS diseases. After being activated from their resting state by microenvironmental stimuli, microglia are considered to primarily exhibit pro-inflammatory and anti-inflammatory states, which is also the focus of this review. In pro-inflammatory states, activated microglia are capable of producing a variety of proinflammatory factors such as tumor necrosis factor (TNF), interleukin-1beta (IL-1β), interleukin-6 (IL-6), interferon-gamma (IFN-γ), nitric oxide (NO), reactive nitrogen intermediates (RNI), reactive oxygen species (ROS) and CC-chemokine ligand 2 (CCL2). They are responsible for controlling the expression of a number of identifiable cell surface markers, majorly histocompatibility complex (MHC) II, integrin alpha-M (CD11b), CD16, CD9, CD10, CD32, CD68 and CD86 (64–66). Pro-inflammatory microglia induce inflammation and neurotoxicity, which can stimulate the conversion of astrocytes into neurotoxic reactive astrocytes A1 (58). Also, they can exacerbate ROS production, axonal injury, and cell death in regions key to cognitive functions such as the cortex, hippocampus and basal ganglia, leading to nerve fiber and neural network abnormalities related to mood and neurocognition (67, 68). In anti-inflammatory states, activated microglia express anti-inflammatory molecules, such as interleukin-10 (IL-10), interleukin-4 (IL-4), tumor growth factor-beta (TGF-β) and neurotrophic factors as well as cell surface markers mannose receptor CD206, CD163, Arginase-1 (Arg-1), transglutaminase-2 (TG2), and YM1 (chitinase-like-3, Chi3l3) (69, 70). Anti-inflammatory microglia inhibit pro-inflammatory microglial functions and promote debris removal, extracellular matrix deposition, axon growth, angiogenesis, and functional synaptogenesis, thereby intensifying tissue repair and remodeling (71–73). In general, microglial dual pro- and anti-inflammatory states open up multiple new perspectives for the treatment of related diseases including IS.

### 2.2 The role of microglia in IS: A double-edged sword

**Figure 2:**
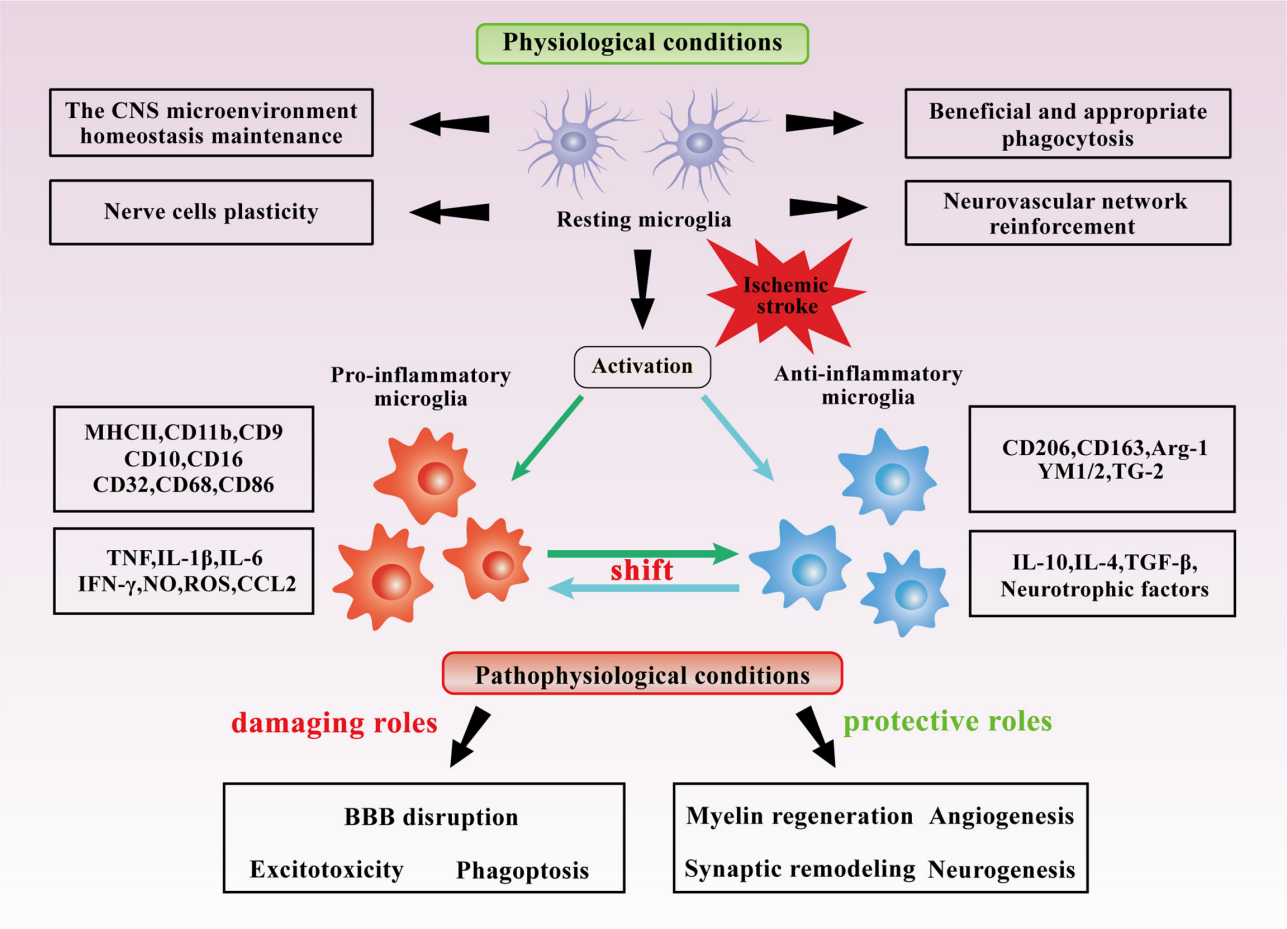
Dual roles of microglia in IS. Under normal conditions, microglia maintain the CNS microenvironment homeostasis and appropriately regulate the neurovascular network. Under the stimulation of cerebral ischemia, microglia are likely to be primarily activated to anti-inflammatory states at the early stages and pro-inflammatory states at the later stages, synthesize and secrete various cytokines and mediators. In IS, Microglia may be beneficial in angiogenesis, myelin regeneration, synaptic remodeling, and neurogenesis in IS, but they may also be detrimental in BBB disruption, excitotoxicity, and phagoptosis.

After ischemic brain injury, microglia are rapidly recruited to the lesion area and further activated. Morphologically, ramified microglia are functionally “resting” or “quiescent,” with long thin processes and small cell bodies, whereas amoeboid microglia are “active,” with shorter processes and larger cell bodies (74, 75). A quantitative spatiotemporal analysis of microglia morphology during IS and reperfusion indicated microglia ramification increased initially in ipsilateral brain regions, whereas progressively de-ramified following IS and 24 hours of reperfusion (76). This may disclose the time point at which microglia transition to an activated state after ischemia-reperfusion (IR). Subsequently, microglia exhibit a spatiotemporally-dependent activation state dominated by pro- and anti-inflammatory states and actively engage in the pathophysiology of IS, as mentioned explicitly in the review by Jiang et al. (65). It was hypothesized that following IS injury, microglia activation states change dynamically, exhibiting predominantly beneficial anti-inflammatory states in the early stages, followed by a subsequent transition to primarily detrimental pro-inflammatory states in the later stages. The role of activated microglia is complicated and could be altered through many complex factors, namely the severity of initial ischemia, the different IS pathological phases and the location within the lesion, etc. The bidirectional and key roles that activated microglia might play in IS are summarized in **Figure 2**. However, because of the spatiotemporal specificity of microglia in IS and the multiple complicated influencing factors, the role of microglia in IS requires further investigation, and the appraisal of the benefits and negatives of the function can also be improved.

#### 2.2.1 The protective roles of microglia in IS

Microglia may exhibit beneficial effects in angiogenesis, myelin regeneration, synaptic remodeling, and neurogenesis after IS.

Extracellular vesicles derived from oxygen-glucose deprivation (OGD) pretreated alternatively activated microglia promote cell viability, migration, and angiogenesis in bEnd.3 cells *via* the TGF-β/Smad2/3 pathway, while IL-4-polarized BV2 microglia cells promote angiogenesis by secreting exosomes to counteract hypoxic injury (77, 78). From previous studies, treatment of human umbilical vein endothelial cells (HUVEC) with conditioned medium collected from berberine (BBR)-treated BV2 cells which were stimulated by lipopolysaccharide (LPS), promoted angiogenesis, but was reversed by adenosine 5-monophosphate (AMP)-activated protein kinase (AMPK) inhibitor (compound C) and AMPK siRNA (79). Conjointly, various cytokines such as VEGF, IL-10, IL-1 and TNF released by microglia were potentially involved in the reconstruction of cerebral blood vessels following IS (80, 81). Lu et al. reported that plx5622-fed mice with significant depletion of microglia/macrophages demonstrated angiogenic impairment as revealed in a double-labeled immunostaining for BrdU and CD31, a marker of the microvascular endothelial cells (EC). Moreover, this study revealed differences in angiogenesis between young and old microglia in the ischemic brain by functional enrichment analysis performed by ingenuity pathway analysis (IPA). According to the study, the young microglia had better functional scores than the aged microglia 14 days after stroke compared to the sham-operated group (82).

Upon a demyelinating injury, oligodendrocyte precursor cells (OPCs), the myelin-forming glial cells enwrapping neuronal axons and ensuring impulse transmission, migrate to the lesion site, proliferate, and differentiate to myelinating oligodendrocytes. However, early ischemic damage affects OPCs, resulting in loss of oligodendrocyte integrity contributes to the axonal degeneration and long-term functional and cognitive deficits after stroke (83, 84). Depletion of microglia could interfere with upregulation of myelination markers on days 3 and 7 post-cerebral ischemia in mice (85). Mediators synthesized by pro-inflammatory or anti-inflammatory/pro-regenerative microglia can effectively promote myelin repair and have shown promising outcomes in the form of extracellular vesicles (EVs) or nanoparticles in studies. Microglia can secrete IL-4, which has been found to directly trigger the differentiation of oligodendrocyte progenitor cells, thereby reinforcing the myelin lipid synthesis by activating the PPARγ signaling pathway (86). The study also showed that IL-4 treatment significantly reduced the ratio of the inner axonal diameter to the total outer diameter of myelinated fiber and increased the number and paranodal length of normal organization of Ranvier (NOR) in the ischemic external capsule. This suggests that IL-4 post-treatment improves the structural and functional integrity of myelinated fibers after stroke. TNF, particularly the transmembrane form (tmTNF) of microglia has also been implicated in promoting oligodendrocyte differentiation and remyelination by interacting with TNF receptor 2 *in vivo* and vitro studies (87–89). Using CAG-eGFP reporter mice to trace the fate of GPR17-expressing OPCs, which labeled by the green fluorescent protein (GFP), the study revealed that infusion of EVs derived from pro-regenerative microglia favored protective microglia/macrophages functions and enhanced the maturation of GFP+ OPCs at lesion borders. Furthermore, after primary OPCs were exposed to IL-4 EVs in the presence of the nonselective TNF blocker etanercept, the percentage of MBP+ cells induced by IL-4 EVs was decreased (90). miR-23a-5p was enriched in pro-regenerative microglial EVs and could promote oligodendrogenesis and myelin repair *in vitro* and *in vivo*, possibly *via* directly targeting Olig3. knocking down miR-23a-5p in pro-regenerative microglial EVs reversed the beneficial effects (91). Iron is required for myelin cholesterol and fatty acid synthesis in oligodendrocytes and microglia are suggested to be a source of extracellular iron for oligodendrocytes by releasing ferritin. Antagonizing the bone morphogenetic protein (BMP) could alter the iron status in reactive microglia from the iron-storing to the iron-releasing phenotype *via* the inhibition of the OGD/R-induced BMPs/hepcidin pathway, contributing to microglial iron release and myelin production in oligodendrocytes (92). The activation of microglial TREM2 signaling pathway mediates the clearance of myelin debris and promotes myelin regeneration (93).

Synaptic dysfunction is one of the pathophysiological events triggered by IS, which eventually leads to neuronal cell death (94, 95). Activation of microglia and their mutual interactions with other cells can be engaged in synaptic remodeling processes (96–98). Microglia regulate the synaptic integrity by actively remodeling the synaptic and perisynaptic environment through complement signaling, but the complement signaling could be disrupted when the brain presents a prolonged microglia activation (99). Adult hippocampal neurons express IL-33 in an experience-dependent manner and can act on the corresponding receptors in microglia. The conditional removal of neuronal IL-33 or microglia IL-33 receptors have been found to lead to a reduction in the dendritic spines, a decrease in the number of new neurons, and impaired accuracy of fear memory extraction. In combination, the neuronal IL-33 directs microglia to phagocytose the extracellular matrix (ECM), and its loss could result in impaired ECM phagocytosis and the accumulation of ECM proteins that accompany synaptic contacts (100). During a critical window of postnatal development in mice, GABA receptive microglia selectively interacted with inhibitory cortical synapses. In this reaction, GABA initiates a transcriptional synaptic remodeling program within these specialized microglia that in turn shapes the inhibitory synaptic connections without affecting excitatory synapses (101). Microglial presynaptic phagocytosis and spine head filopodia induction, as well as SIRPα signaling can also regulate synaptic remodeling (37, 102, 103).

Reactive astrogliosis and glial scar formation are among the primary factors causing difficulty in achieving neurogenesis and functional recovery after IS (104, 105). At the later stage of IS, overproliferation of reactive astrocytes could form glial scar, which secretes chondroitin sulfate proteoglycan in response, inhibit axonal regeneration, thus restraining neurogenesis and the recovery of neurological function (106, 107). Studies have demonstrated that small extracellular vesicles derived from anti-inflammatory microglia reduced the astrocyte proliferation gene signal transducer, lowered the activator of transcription-3, decreased the glial fibrillary acidic proteins, and inhibited astrocyte proliferation *via* the miR-124/STAT3 signaling pathway. In addition, anti-inflammatory microglial small extracellular vesicles treatment reduced Notch 1 expression and improved Sox2 expression in astrocytes, whereby promoting the transformation of astrocytes into neuronal progenitors in ischemic mice (104). Interestingly, several studies have reported that glial scar formation also contributes to the regeneration of central neural axons, reflecting the dual role of activated microglia in the ischemic microenvironment of the brain (108–110).

#### 2.2.2 The harmful effects of microglia in IS

On the contrary, microglia may play damaging roles in BBB disruption, excitotoxicity and phagoptosis after IS.

The BBB is a highly specialized structure formed by EC through interaction with pericytes, astrocytes, neurons, and microglia of the neurovascular unit (NVU), which separates the components of blood circulation from neurons and maintains the chemical compositions of the neuronal “environment”. This helps to ensure the proper function of some crucial activities such as neuronal circuits and synaptic transmission, which depends on a closely monitored neurovascular environment. However, BBB disruption due to breakdown of tight junctions, vascular degeneration, inadequate cerebral perfusion and inflammatory responses can further worsen the vicious cycle of related pathological processes (111, 112). Microglia populate the brain and are localized near the cerebral microvascular system prior to the development of the cerebral vascular network, while the resting microglia contribute to the maintenance of the BBB phenotype. Studies on co-cultures of mouse brain EC with resting microglia revealed an enhanced endothelial expression of the key tight junction proteins occludin and ZO-1, and *in vivo* studies demonstrated that perivascular microglia may directly stimulate the key proteins including claudin-5 to assemble the BBB endothelium into tight junction complexes (113). After ischemia/reperfusion (I/R) injury, microglia induce necroptosis of EC with evidence that EC co-cultured with microglia after oxygen-glucose reperfusion (OGD/R) treatment exhibited the typical morphological features of necroptosis, as it displayed translucent cytoplasm, swollen mitochondria, enlarged cell volume, and ruptured cell membranes. Pro-inflammatory microglia induce necroptosis of ischemic cortical vascular EC by secreting TNF. Nonetheless, this condition was effectively alleviated by Infliximab, a human and mouse chimeric monoclonal antibody that specifically blocks TNF. Also, inhibition of NF-κB nuclear translocation in microglia reduced endothelial necroptosis under OGD/R, and treatment with an NF-κB inhibitor BAY11-7082 decreased NF-κB nuclear translocation by approximately 50% in microglia affected by OGD/R, and TNF release in microglia (114). Furthermore, investigations have revealed that the relationship between microglia and blood vessels became increasingly tighter with the duration of ischemia, as exhibited by an increase in the number of perivascular microglia and a decrease in the number of blood vessels. Microglia population form perivascular clusters and engulf all or part of the adjacent vascular endothelium, leading to vascular disintegration and BBB breakdown. Extravasating serum proteins such as albumin and fibrinogen further recruit and activate microglia, thus increasing the damage done to the BBB (115). For the treatment of cerebral ischemia with tissue-type fibrinogen activator thrombolysis, the integrin Mac-1 expressed on microglia acts together with the endocytic receptor LRP1 in the NVU to promote tPA-mediated PDGF-CC activation, thereby increasing cerebrovascular permeability and intra-cerebral hemorrhage during IS (116).

Excitotoxicity, a specific type of neurotoxicity mediated by glutamate and caused by over-stimulation of neuronal glutamate receptors, provides a link between ischemia and neuronal death. It also helps by intervening in the mechanisms associated with excitotoxicity in IS necessary for controlling stroke damage (117, 118). IS induces neuroinflammation and triggers the release of proinflammatory cytokines, which causes excitotoxicity, leading to neuronal dysfunction, and eventually cellular death. Proinflammatory cytokines released by activated microglia such as IL-2, INF-γ, or TNF boost the activation of the tryptophan- and serotonin-degrading enzyme indoleamine 2,3-dioxygenase (IDO) and its subsequent enzyme kynurenine monooxygenase. These cytokines have also been studied to enhance the production of quinolinic acid, a strong agonist of the glutamatergic N-methyl-D-aspartate (NMDA) receptor, promoting synaptosomal glutamate release and supported the release of astroglial glutamate and d-serine (119, 120). In addition, pro-inflammatory factors such as TNF and INF-γ directly increased glutamate synthesis and decreased glutamate uptake in microglia through signals involving protein kinase C, cAMP responsive element-binding protein and CAAT-enhancer-binding protein-beta. For astrocytes, the same effects were achieved by inhibiting and reversing astroglial excitatory amino acid transporters (121–123). Furthermore, activated neuroinflammatory microglia induce A1 astrocytes which makes them lose their ability to promote neuronal survival, growth, synapse formation and phagocytosis, and induce neuronal and oligodendrocyte death by secreting cytokines IL-1α, TNF and C1q. When the formation of A1 astrocytes is blocked, the death of axonically severed CNS neurons *in vivo* is prevented (124). Excessive glutamate binds to glutamate receptors, such as NMDA receptors and dl-alpha-amino-3-hydroxy-5-methyl-4-isoxazole propionic acid (AMPA) receptors. This causes increased influx of Na^+^ and Ca^2+^ ions into the cell, which is a major mechanism of secondary damage and neuronal death after stroke (125, 126).

Microglia have been studied to be involved in phagoptosis, a process in which phagocytosis of live cells results in death of the engulfed cells (127, 128). Inflammation in IS induces excessive accumulation of intracellular Ca^2+^, and results in the activation of phospholipid scramblase TMEM16F and inactivation of the aminophospholipid translocase ATP11C/ATP8A in neurons. In this way, stressed-but-still-viable neurons are induced in the penumbra to expose the “eat-me” signal phosphatidylserine (PS). Neurons exposed to PS can be recognized by the opsonin MFG-E8 and vitronectin receptors on microglia or by the opsonin Gas6 and MerTK (Mer receptor tyrosine kinase) receptors on microglia, triggering primary microglia phagocytosis. Notably, the study established that in the absence of activated microglia, stressed neurons could return to a normal state and survive longer. As an evidence, a flow cytometry analysis showed a significant decrease in the proportion of neurons exposed to PS and a growth in the proportion of healthy cells after 24 hours of reperfusion compared to 12 hours of reperfusion, while cell death did not increase. Furthermore phagocytosis of neurons was delayed by at least 72 h, suggesting that there could be a therapeutic target in the time course between the neuronal PS exposure and the initiation of primary phagocytosis (129). Wang et al. discovered that the b-series ganglioside GD3 and its biosynthetic enzyme GD3 synthase were predominantly upregulated in mouse hippocampal microglia between 2 - 7 days after total cerebral ischemia, and the knockdown of GD3 delayed the phagocytosis toxicity of microglia to neurons (130). Additionally, during the post-stroke repair and remodeling phase, reactive microglia and astrocytes activated phagocytose synapses *via* MEGF10 and MERTK-related pathways, thereby impeding brain repair. Accordingly, inhibiting the glial cell phagocytosis of synapses increased pre- and postsynaptic levels, and the number of dendritic spines, thus improving neurobehavioral outcomes in mice with IS (108). In this section, we discussed only a part of the damaging effects of microglia-mediated phagocytosis on brain tissue. Definitely, there are yet other numerous favorable aspects of microglia phagocytosis which are mentioned in other sections. An example is observed in the process by which microglia control neutrophil accumulation in the injured brain and prevent their damage to blood vessels and tissues (131). Specifically, efferocytosis, or phagocytosis of dead/dying cells by brain-resident microglia contributes to the regression of inflammation after stroke (132).

## 3 Microglia autophagy in ischemic stroke

### 3.1 Autophagy

In the 1860s, the term “autophagy” was first proposed when some researchers observed that cells enclose their intracellular components in membranes to form sac-like structures and transport them into a small compartment known as “lysozyme” (133, 134). With the advances in research, autophagy was reported as a stress-adaptive process induced by autophagy-related genes (ATG), which mainly occurs in eukaryotic cells (135, 136). Overall, through non-selectively degrade cellular components in bulk and selectively eliminate and secrete specific cytoplasmic contents, autophagy is responsible for modulating the development, differentiation, and death of cells and tissues, and maintaining inflammation and immune homeostasis, adapting to metabolic demands, etc (137–141). Autophagy has been found to be involved in various human diseases such as liver disease, lung disease, and neurodegenerative diseases (142–146). It can be triggered by various stress conditions including starvation, hypoxia, oxidative stress, protein aggregation, endoplasmic reticulum (ER) stress, and mammalian target of rapamycin (mTOR) inhibitors (139, 147). Generally, autophagy in mammalian cells is divided into microautophagy, macroautophagy and molecular chaperone-mediated autophagy (148–152). Among these, macroautophagy is the most studied type and widely recognized autophagy in mammalian cells, hence we focused mainly on macroautophagy in this review.

The outline macroautophagy, hereafter referred to as “autophagy”, process has been clearly sketched, involving a series of sequential steps. First is the occurrence of phagophore. After receiving autophagy-inducing signals, a small “liposome”-like membrane structure is formed somewhere in the cytoplasm. Next, the membrane continuously expands to form a flat 2-layer lipid membrane, named phagophore, which is one of the direct pieces of evidence that autophagy occurred. Stress signals activate the ULK1-ATG13-FIP200 complex including ULK1, ATG13, RB1CC1/FIP200, ATG101, and then the ULK1-ATG13-FIP200 complex triggers nucleation of the phagophore by phosphorylating components of the class III phosphatidylinositol 3-kinase (PtdIns3K) complex including BECN1/Beclin 1, PIK3C3/VPS34, PIK3R4/VPS15, ATG14, NRBF2, UVRAG and possibly other factors. Therefore, the local phosphatidylinositol-3-phosphate (PI3P) production is activated at a characteristic ER structure called the omegasome (139). The second step is characterized by the formation of autophagosome. Phagophore is continuously stretched to incorporate some components and ultimately develops into a spherical double-membrane structure, namely the autophagosome, concurrently capturing misfolded proteins or damaged organelles into autophagosomes randomly or selectively, such as misfolded proteins are ubiquitinated and linked to the p62 protein, which is then transported to the autophagosome. In the above processes, PI3P interacts with the WIPI2, which recruits the ATG12-ATG5-ATG16L1 complex that promotes the conjugation of ATG8 family proteins LC3 and GABARAP subfamilies and phosphatidylethanolamine (PE). The conjugation thus leads to the membrane binding of lipidated forms including the conversion of LC3-I into LC3-II, which is the characteristic signature of autophagic membranes. Regarding the elongation of the autophagosomal membrane, ATG9 vesicles, ATG12–ATG5–ATG16L1 and LC3/ATG8-PE ubiquitin-like binding systems are all involved (139, 144, 153). Next, the autophagosomes fuse with lysosomes to form autophagolysosomes. Lastly, the autophagic cargo is degraded by lysosomal enzymes, and the salvaged nutrients including amino acids, fatty acids, etc., are transported into the cytoplasm for recycling, and the residues are excreted out of the cell (139, 154). Recently, researchers demonstrated that autophagy involves a wide range of signal regulatory pathways which are mainly divided into mTOR-dependent pathways and mTOR-independent pathways. apart from the classical mTORC1 pathway, several mTOR-independent autophagy pathways, such as Ca^2+^, AMPK, MAPK/JNK (c-Jun N-terminal kinase), ROS, hypoxia-inducible factor-1 alpha (HIF-1α), miRNA and the transient receptor potential calcium ion channel (TRPML), forming a sophisticated and complicated network of signals that regulate autophagy positively or negatively (155).

Increasing evidence indicates that autophagic response is closely related to brain pathology after IS (156). Genome-wide association studies performed for SVO stroke identified that the novel risk loci for SVO stroke contained genes, particularly ATG7, which play crucial role in autophagy formation (157). Autophagy-related gene microarray analysis and bioinformatics analysis conducted on peripheral blood samples obtained from IS patients and controls revealed autophagy take part in IS-induced injuries, and mitochondrial autophagy played a crucial role in this process (158). Microglia are involved in a variety of physiopathologic processes of IS, most notably in the initiation and maintenance of neuroinflammation. Exploring the role of microglia autophagy in inflammation responses following IS can also provide new insights for the research and treatment of the disease.

### 3.2 Microglia autophagy and inflammation in ischemic stroke

During IS, the deprivation of oxygen and glucose caused by impaired cerebral blood supply leads to a series of damaging effects such as oxidative stress, excitotoxicity, and inflammation. Ischemia is one of the most important forms of energy deprivation and often leads to cellular autophagy. Under nutrient-rich condition, autophagy is inhibited by the mammalian target of mTOR. Meanwhile, high mTOR activity prevents ULK1 activation by phosphorylating ULK1 Ser 757 and disrupting the interaction between ULK1 and AMPK (159). Contrarily, under energy-shortage conditions, AMPK, which functions as a cellular energy sensor, is activated through an increase in AMP/ATP or ADP/ATP ratios under energy stress. This in turn resists cerebral ischemic injury by switching on catabolic pathways and switching off ATP-consuming processes (160). On the one hand, AMPK directly phosphorylates the mTOR upstream regulator TSC2 on Thr1227 and Ser1345, and the mTORC1 subunit RAPTOR on Ser722 and Ser792, thereby reducing mTOR activities and relieving the inhibitory phosphorylation on ULK1 to activate autophagy (161). On the other hand, AMPK promotes autophagy by directly activating ULK1 through phosphorylation of Ser 317 and Ser 777 (159). Hypoxia activates IκB kinase (IKK) and phosphorylates IκBa, releasing NF-κB and transferring it to the nucleus to mediate autophagy initiation *via* the p53 signaling pathway. Hypoxia, on the other hand, stimulates the release of HIF-1 and the expression of BNIP3, and thus mediate the dissociation of beclin-1 and Bcl-2. ULK1 activation and the release of beclin-1 promotes the formation of PtdIns3K complexes and the ULK1-ATG13-FIP200 complexes. Excitotoxicity prevents the fusion of autophagosomes and lysosomes, thereby disrupting autophagic flux (153).

Autophagy and inflammation are two intertwined processes pivotal for microglia to perform their functions in IS, and their fine-tuned interplay is complex. Under normal circumstances, autophagy is suggested to act as a brake to suppress inflammation in microglia, while under the activated conditions, autophagy may function as both a “brake and accelerator” pedal in ischemia inflammation. This is demonstrated *in vitro* cellular and *in vivo* animal models of IS. Microglia autophagy was induced in the early stages of OGD/R, and both pro-inflammatory and anti-inflammatory microglia increased; however, in the late stages of OGD/R (72 hours), microglia autophagy was inhibited, pro-inflammatory microglia increased, and anti-inflammatory microglia decreased (162). In mice, permanent middle cerebral artery occlusion (pMCAO) for 12 hours, 48 hours, or 72 hours induced microglia autophagy, which contributed to ischemic inflammatory response (163). However, how microglia autophagy interacts with neuroinflammation in the subsequent time course of IS remains to be fully explored *in vivo* and *in vitro* models. Simultaneously, the mechanism by which their interactions can be effectively regulated to reduce the damage to post-ischemia brain tissue is a question that is yet to be answered.

#### 3.2.1 Microglia autophagy regulates inflammatory responses in ischemic stroke

Following IS, the altered expression of numerous microglia molecules is actively involved in the regulation of microglia autophagy and the inflammatory response. Focusing on the spatiotemporal changes of these molecules in microglia and their mediated autophagic responses in cerebral ischemic inflammation, as well as therapeutically targeting these molecules, may point to other potential directions towards the clinical treatment of IS.

Multifarious endogenous molecules are vital for the induction of microglia autophagy to negatively or positively regulate ischemic inflammatory responses. Phosphodiesterase enzyme 1-B (PDE1-B) expression in the microglia was progressively elevated in in OGD-treated BV2 cells and in the peri-infarct cortex of mice 3 days and 14 days after MCAO (164). With OGD treatment, PDE1-B knockdown or inhibition significantly increased auphagy levels and blocked the increase in CD11b expression as well as the decrease in Arg-1 and Iba-1 expression in BV2 cells. The use of an autophagy inhibitor, 3-MA, significantly inhibited such effect. After a photothrombotic stroke in mice, the level of poly (ADP-ribose) polymerase family member 14 (PARP14) was significantly increased in the peri-infarct region on days 1, 3, 7, and 14 (165). In OGD-treated BV-2 cells and primary mouse microglia, PARP14 promoted the expression of MAP1LC3B-II mainly by enhancing autophagic flux rather than blocking lysosomal processing of autophagosomes, which constricted microglia activation by suppressing transcription of the lysophosphatidic acid receptor 5 (LPAR5) gene. PPARγ coactivator-1α (PGC-1α), a master co-regulator of gene expression in mitochondrial biogenesis, was transiently upregulated in microglia after acute ischemic stroke (AIS). In comparison with the control group without neurological diseases, PGC-1α expression in microglia increased on day 1 after the onset of stroke but decreased in patients who died between days 3-10 after stroke onset (166). Correspondingly, mRNA and protein levels of PGC-1α in mouse microglia also peaked on day 1, but gradually decreased from day 3 to day7 after tMCAO. Microglia PGC-1α overexpression promoted autophagic and mitophagic activities *via* ULK1, inhibited NLRP3 activation and subsequently reduce the production of proinflammatory cytokines, and constrained neurological deficits. HIF-1α is highly expressed under hypoxic conditions and heterodimerizes with HIF-1β to form HIF-1, then translocates to the nucleus (167). HIF-1 regulates the expression of its downstream targets BNIP3 and BNIP3L, which in turn induce autophagy by disrupting the interaction of Beclin1 with Bcl-2 and Bcl-XL and suppressing microglia inflammatory cytokine overexpression through autophagy induction during early hypoxia (167, 168). Inhibition of the serine/threonine kinase GSK-3β suppressed the release of inflammatory cytockines such as IL-1β, TNF and increased autophagy activation in pMCAO rats and n the cultured microglia (169). DJ-1 interference combined with a Sirt1 inhibitor increased the effect of DJ-1 interference on microglial polarization from anti-inflammatory to pro-inflammatory states, decreased the level of the Atg5-Atg12-Atg16L1 complex, and inhibited autophagy during cerebral I/R injury (170). Activation of autophagy *via* the AMPK pathway could facilitate microglial anti-inflammatory activation and exert neuroprotective effects during an inflammatory process (171–175). P2X7 receptor can activate AMPK pathways in the regulation of mitochondrial and lysosomal functions in microglia (176, 177). However, certain pathways including the AMPK pathway in microglia autophagy in the regulation of inflammation in IS remains poorly understood.

Induction of microglia autophagy, on the other hand, aids in ischemic inflammatory responses. Protein tyrosine phosphatase 1B (PTP1B) levels in the ipsilateral cerebral cortex of rats showed a significant increase at 12 hours after IR injury (178). A peak observed at 24 hours and a decrease after 72 hours compared with sham-operated controls, with an upregulation of PTP1B expression after rat IR injury was prominent in microglia. Microglia PTP1B expression was significantly elevated after IR injury, which may increase ER stress-dependent primary microglia autophagy by specifically promoting the PERK signaling pathway. By that, OGD/R-induced microglia activation and inflammatory responses were exacerbated. Microglia upregulated sphingosine kinase 1 (SphK1) after IR injury. SphK1 induced microglia autophagy *via* the tumor necrosis factor receptor-associated factor 2 (TRAF2) pathway and promoted microglia excessive activation (179). Sphingosine-1-phosphate (S1P), a pleiotropic bioactive sphingolipid metabolite, is synthesized by SphK1. GPR37, also known as the parkin-associated endothelin-like receptor (Pael-R), is an orphan G protein-coupled receptor expressed in the CNS. At 6 and 12 h after focal IS in GPR37 knockout (KO) mice, the LC3II-LC3I ratio was significantly increased compared to WT mice. At 12 hours after stroke,the mTOR signaling pathway of GPR37 KO mice was greatly inhibited. In addition, the number of Iba-1-positive cells in the ischemic cortex of GPR37 KO mice and the mRNA levels of pro-inflammatory mediators was dramatically higher than was observed for WT mice. However, in this study, whether GPR37 induces autophagy in microglia is unknown (180). Neural S1P accumulation mediated microglia autophagy defects and mediated IL-6 release release (181). TLR4 could function as an upstream channel of multiple signaling pathways for autophagy (inhibition or initiation)-induced pro-inflammatory activation in microglia (182–184). During cerebral ischemia, TLR4 promoted ischemia-induced microglia autophagy, as evidenced by an increased LC3II and beclin-1 and a decrease in p62 *in vitro* and *in vivo*. In addition, TLR4-mediated microglia autophagy was involved in microglial pro-inflammatory activation, as demonstrated by the upregulation of pro-inflammatory factors, and incubation with autophagy inhibitors wortmannin (Wm) or bafilomycin A1 (Baf) blocked microglia pro-inflammatory polarization (185).

**Figure 3:**
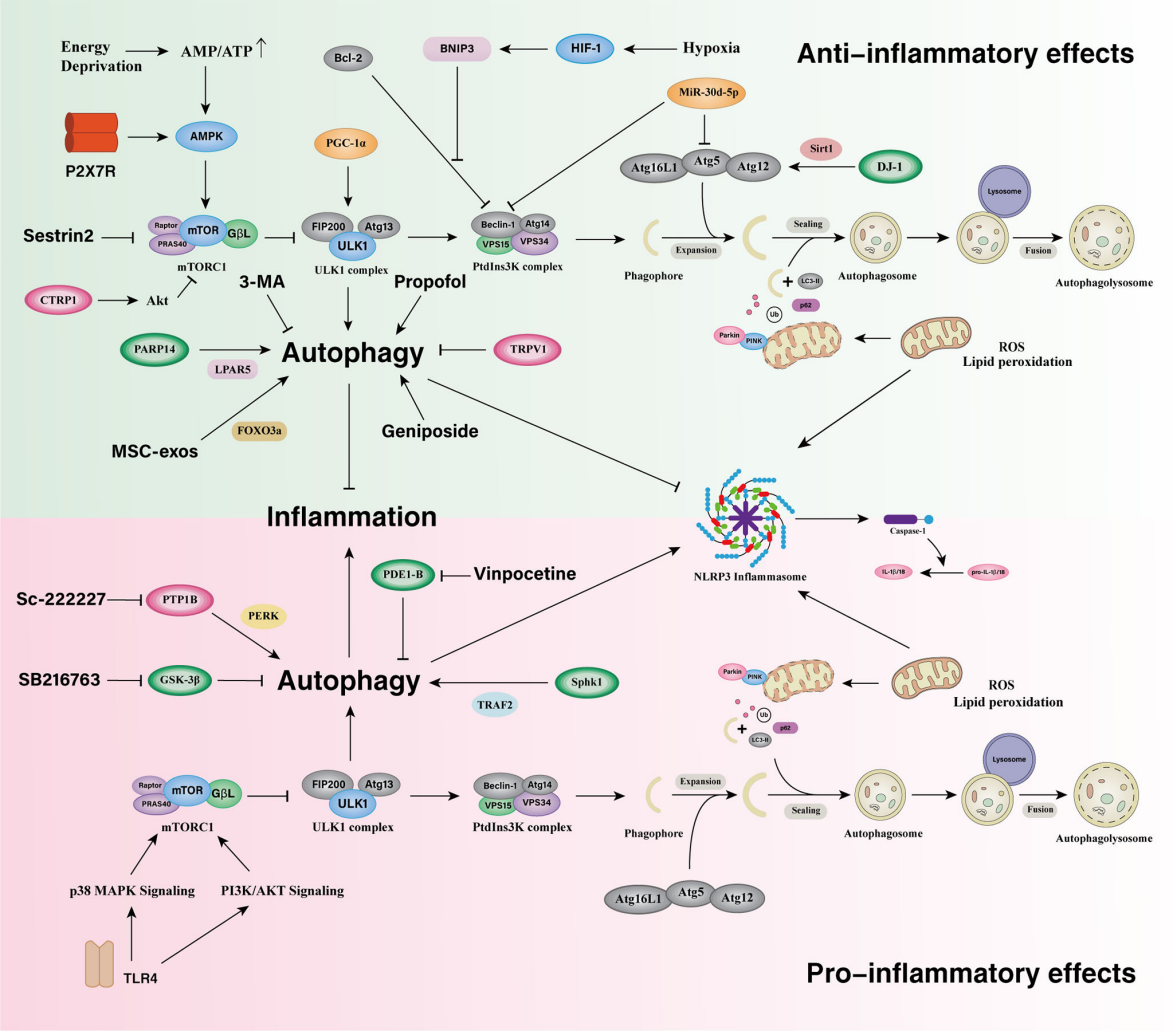
Microglia autophagy in the regulation of inflammatory responses in ischemic stroke. Microglial autophagy bidirectionally regulates inflammatory responses and there are complex interactions between them in ischemic stroke. The downregulation of Microglial PDE1-B, GSK-3β, and the upregulation of PARP14 promote microglia autophagy and suppress inflammatory responses. The upregulation of PGC-1α promotes autophagy and mitophagy *via* ULK1 and inhibits the overactivation of NLRP3 inflammasome. DJ-1 activates the Atg5-Atg12-Atg16L1 complex *via* Sirt1 and alleviates inflammatory responses. HIF-1 regulates the expression of BNIP3, induces autophagy by disrupting the interaction of Beclin1 with Bcl-2 and suppresses microglia inflammatory cytokine overexpression during early hypoxia. Activation of autophagy *via* the AMPK pathway can facilitate microglia alternatively activation and exert neuroprotective effects during an inflammatory process. P2X7 receptor can activate AMPK pathways in the regulation of mitochondrial and lysosomal functions in microglia. Sestrin2 promote microglia autophagy *via* inhibiting mTOR pathway to attenuate microglial inflammatory response. The upregulation of TRPV1 and CTRP1 inhibit autophagy and reduces the inflammatory response. MiR-30d-5p inhibits Beclin-1 and ATG5 expression and exerts an anti-inflammatory effect. Microglial PTP1B, SphK1, and TLR4 induce autophagy and promote microglial proinflammatory activation.

Several molecules, in turn, mitigate the inflammatory response of cerebral ischemia by inhibiting microglia autophagy. Transient receptor potential vanilloid 1 (TRPV1), a non-selective cationic channel expressed in microglia, suppressed NLRP3 inflammasome by impairing OGD/R-induced microglia autophagy, culminating in an anti-inflammatory effect (186). After AIS, microglial miR-30d-5p was observed to be downregulated. MiR-30d-5p significantly inhibited Beclin-1 and ATG5 expression at the mRNA level by targeting their 3’UTRs. Exosomes from ADSCs overexpressing miR-30d-5p inhibited microglia polarization to proinflammatory activated states by reversing the OGD-induced autophagy (187). C1q/tumor necrosis factor-related protein-1 (CTRP1), a newly identified adiponectin paralog that modulates metabolism and inflammation, was dramatically upregulated in serum in stroke patients and in BV2 microglia exposed to OGD/R. CTRP1 mRNA and protein levels in BV2 microglia increased gradually over 3 hours, 6 hours, 12 hours, and 24 hours after 3 hours of OGD, peaking at 3 hours of OGD and 12 hours of reperfusion. Recombinant CTRP1 or CTRP1 overexpression resulted in decreased OGD/R-induced autophagy as well as diminished concentrations of proinflammatory cytokines in in BV2 cells. Furthermore, recombinant CTRP1 increased the phosphorylation of Akt and mTOR in BV2 cells, and inhibition of Akt with A6730 reversed the inhibitory effect of recombinant CTRP1 on BV2 cells autophagy and inflammation response (**Figure 3**) (188).

In general, more existing research has been conducted on the induction of autophagy than on the inhibition of autophagy in microglia after cerebral ischemia. Microglia autophagic responses in IS may primarily negative regulators of inflammatory responses in microglia following cerebral ischemia. It should be noted that research on the spatiotemporal relationship of post-stroke microglial autophagy to inflammation regulation is relatively insufficient. Furthermore, the underlying regulatory mechanisms of various autophagy pathways for inflammatory responses in microglia are not well understood.

#### 3.2.2 Treatment of ischemic inflammatory response targeting on microglia autophagy

Previous studies have targeted microglia autophagy to control the inflammatory response after ischemic stroke, which could be a potential strategy to promote tissue repair and improve functional outcomes of stroke. In addition to endogenously targeting those above-mentioned microglia molecules, some exogenous treatments have been demonstrated to be constructive for microglial autophagy to regulate inflammatory responses (**Table 1**). PDE1-B inhibition by vinpocetine substantially enhanced microglia autophagy, which contributed to microglia polarization to anti-inflammatory states under ischemic conditions. In addition, exosome derived from OGD-BV2 causes neuronal damage whereas exosomes derived from vinpocetine-treated BV2 protects the neurons against stroke-induced damage *in vitro* and *in vivo* (164). The GSK-3β inhibitor SB216763 activated autophagy and suppressed inflammatory response in microglia. Contrarily, inhibition of autophagy by Beclin1-siRNA increased inflammatory response in the SB216763-treated microglia (169). The PTP1B inhibitor sc-222227 reduced ER stress-dependent primary microglia autophagy *via* the PERK signaling pathway and alleviated OGD/R-induced microglia activation and inflammatory responses (178). Mesenchymal stem cell-derived exosomes (MSC-exos) enhanced microglia mitophagy to inhibit OGD/R-Induced inflammatory response by upregulating FOXO3. In OGD/R-exposed BV-2 cells MSC-exos increased cell viability and the expression of TOM20 and COX IV while decreasing the expression of NLRP3, cleaved caspase-1, and the release of IL-1 and IL-18. Furthermore, 3-MA, mdi-1, and FOXO3a siRNA hindered the effect with MSC-exo treatment (189). Exogenous sestrin2 drove microglia to the anti-inflammatory states by inhibiting the mTOR signaling pathway and restoring autophagic flux during experimental brain ischemia (190). Anesthetic medication propofol treatment inhibited the IL-6 and TNF levels but promoted IL-10 and IL-4 expression in OGD-activated microglia. Moreover, propofol treatment increased LC3II/I,Beclin-1, and Atg-7 expression in microglia (191). The classical autophagy inhibitor 3-methyladenine (3-MA) suppressed inflammatory response by lowering microglia autophagy in IS (163, 193). NLRP3 inflammasome is a key mediator in IS-induced neuroinflammation. Following cerebral ischemia, microglia molecules can act as upstream regulatory signals, actively participating in the negative regulation of the NLRP3 inflammasome *via* microglia autophagy. Several drugs are also based on modulating this pathway to alleviate the detrimental effects after cerebral ischemia. By inhibiting NLRP3 inflammasome activation in BV-2 microglia, geniposide, a pharmacologically active com-pound purified from the Chinese herbs *Gardenia jasminoides* Ellis, was found to increase post-OGD/R autophagic activities and thus lower inflammatory cytokine levels (192). In general, there are still relatively few drug studies focusing on microglia autophagy in the treatment of ischemic inflammation, and further drug screening strategies based on microglia autopgagy may provide new direction for research. More properly designed *in vivo* animal experiments may bridge the gap between pre-clinical studies and human clinical trials.

**Table 1:** Mechanisms and effects of microglia autophagy regulators in the regulation of inflammatory responses in IS.

Therapeutic candidate	Target	Mechanisim	Effect	Model	Reference (s)
**Autophagy induction**					
Vinpocetine	PDE1-B	Inhibit PDE1-B to enhance microglia autophagic flux and thus inhibit microglial proinflammatory role.	neuroprotective	MCAO	(164)
	PARP14	Promote microglia autophagy and constrict inflammatory response *via* LPAR5	neuroprotective	photothrombosis OGD	(165)
	PGC-1α	Promote microglia autophagy *via* ULK1 and inhibit the overactivation of NLRP3 inflammasome	neuroprotective	tMCAO	(166)
SB216763	GSK-3β	Inhibit GSK-3β to suppress inflammation by activating autophagy in microglia	neuroprotective	pMCAO	(169)
	DJ-1	Activate the Atg5-Atg12-Atg16L1 complex through Sirt1 to promote microglia autophagy thus playing an anti-inflammatory role	neuroprotective	MCAO/R	(170)
MSC-exos	FOXO3a	Enhance microglia mitophagy to inhibit inflammatory response by upregulating FOXO3	neuroprotective	OGD/R	(189)
Sestrin2	mTOR pathway	Promote microglia autophagy *via* inhibiting mTOR pathway to attenuate microglial inflammatory response	neuroprotective	tMCAO OGD	(190)
Propofol		Promote microglia autophagy and inhibit inflammatory cytokine secretion	neuroprotective	2VO OGD	(191)
Geniposide		Increase autophagic activity and inhibit NLRP3 inflammasome activation in microglia	neuroprotective	OGD/R	(192)
**Autophagy inhibition**					
Sc-222227	PTP1B	Inhibit PTP1B to reduce ER stress-dependent autophagy *via* the PERK pathway and alleviate microglia activation and inflammatory responses	neuroprotective	MCAO OGD/R	(178)
	TRPV1	Inhibit autophagy to suppress NLRP3 inflammasome in microglia	neuroprotective	OGD/R	(186)
	MiR-30d-5p	Suppress microglia autophagy by inhibiting Beclin-1 and ATG5 expression and reduce inflammatory response	neuroprotective	MCAO OGD	(187)
	CTRP1	Attenuate microglia autophagy and inflammatory response by regulating the Akt/mTOR pathway	neuroprotective	pMCAO OGD/R	(188)
3-MA		Inhibit autophagy to suppress inflammatory response in mcroglia	neuroprotective	pMCAO	(163, 193)

## 4 Conclusion

IS is one of the most important causes of neurological morbidity and mortality worldwide. As research progresses, more novel ideas are potentially beneficial in the prevention and treatment of IS. Increasing evidence suggests that microglia is a double-edged sword in IS, as they not only aggravate secondary brain injury but also advantageously ameliorate brain recovery based on different stages of pathogenesis and extracellular microenvironment. Microglia-mediated inflammation is considered to play an important role in the consequent pathogenesis of IS, likewise microglia autophagy. Microglia autophagy are likely to primarily negatively regulate inflammatory responses in IS. However, due to complex microenvironmental factors and the time course of disease progression, microglia autophagy and inflammatory responses are not necessarily always antagonistic in IS. Overall, microglia autophagy is suggested to act as a “brake and accelerator” pedal in ischemic inflammation. Consequently, it is vitally important to find a fulcrum in the complex molecular signal transduction networks of both. So far, relatively few studies have addressed the involvement of microglia autophagy in cerebral ischemic inflammatory responses. Understanding their reciprocal interaction in different phases of stroke may generate new directions for stroke research, as well as promote stroke treatment strategies innovations. In future research, the development of new drugs or the use of existing drugs to target microglia autophagy in IS inflammatory responses may potentially highlighting novel avenues for IS research and therapeutic intervention.

## Author contributions

All authors contributed significantly to the drafting and editing of this manuscript. LP, GQH, and QY conceived the manuscript idea and wrote the manuscript. LY, AW, BL, JD, and JW revised the manuscript content. GSH, ZH, and XZ created the manuscript tables and figures. All authors contributed to the article and approved the submitted version.

## Funding

This study was supported by Grants from the National Natural Science Foundation of China (No. 81903829), the Sichuan Science and Technology Program (No. 2022YFH0115, 22ZDYF3784, and 21RCYJ0021), the Macao Science and Technology Development Fund of Macao SAR (Nos. SKL-QRCM(MUST)-2020-2022 and MUST-SKL-2021-005), the Southwest Medical University (Nos. 2021ZKZD015, 2021ZKZD018, and 2021ZKMS046), the Joint project of Luzhou Municipal People's Government and Southwest Medical University, China (No. 2020LZXNYDJ37), and the Sichuan University and Luzhou Municipal People's Government Strategic cooperation projects (No. 22CDLZYF0027).

## Acknowledgments

This study was supported by Sichuan Key Medical Laboratory of New Drug Discovery and Druggability Evaluation and State Key Laboratory of Quality Research in Chinese Medicine, Macao.

## Conflict of interest

The authors declare that the research was conducted in the absence of any commercial or financial relationships that could be construed as a potential conflict of interest.

## Publisher’s note

All claims expressed in this article are solely those of the authors and do not necessarily represent those of their affiliated organizations, or those of the publisher, the editors and the reviewers. Any product that may be evaluated in this article, or claim that may be made by its manufacturer, is not guaranteed or endorsed by the publisher.
